# Cloning, bioinformatics analysis, and expression of the ubiquitin 2 (ubq-2) gene from the dog roundworm *Toxocara canis*

**DOI:** 10.3389/fvets.2025.1550489

**Published:** 2025-03-31

**Authors:** Pengchen Zhu, Xin Hu, Jiali Huang, Lidan Wang, Zhao Wang, Ruixi Wang, Xinyi Fan, Guoshan Wei, Qin He, Zhaoruiyi Li, Xuan Zhou, Hui Wang, Yue Xie

**Affiliations:** ^1^Department of Parasitology, College of Veterinary Medicine, Sichuan Agricultural University, Chengdu, China; ^2^Beijing Key Laboratory of Captive Wildlife Technologies, Beijing Zoo, Beijing, China; ^3^College of Chemistry and Life Science, Chengdu Normal University, Chengdu, China

**Keywords:** toxocariasis, *Toxocara canis*, UBQ-2, bioinformatics analysis, serodiagnosis

## Abstract

Toxocariasis, caused by the dog roundworm *Toxocara canis,* is a globally distributed zoonotic parasitic disease that poses a significant threat to veterinary and public health. The ubiquitin plus an L40 ribosomal protein (UBQ-2) in parasites plays a crucial role in protein degradation and meiotic divisions, thereby affecting parasite development, survival, and parasite–host interactions. In this study, we identified for the first time a full-length cDNA encoding the UBQ-2 protein from the *T. canis*-omic dataset, called Tcubq-2. After cloning and sequencing, we conducted sequence analysis and structural modeling of TcUBQ-2 using online bioinformatics tools. The recombinant TcUBQ-2 protein (rTcUBQ-2) was prokaryotically expressed and subjected to Western blot analysis to assess its antigenicity and immunoreactivity. Additionally, we performed immunolocalization of the endogenous protein in adult *T. canis* and evaluated its serodiagnostic potential using sera from naïve and infected mice and dogs. Our results showed that the complete cDNA sequence of Tcubq-2 was 387 bp in length and encoded a polypeptide of 128 amino acids, lacking both an N-terminal signal sequence and a transmembrane domain. Sequence and phylogenetic analyses showed that TcUBQ-2 shared the closest genetic distance with its homologs in *Parascaris univalens* and *Ascaris suum*. Real-time quantitative PCR and Western blotting revealed an expression peak of TcUBQ-2 in the intestine-hatched second-stage (L3) larvae compared to other developmental stages of *T. canis*. Tissue localization of endogenous TcUBQ-2 revealed its broad distributions in the body wall, muscle, gut epithelium, and microvilli of adult *T. canis,* with gender-specific expression in the uterus, ovary, and non-embryonated eggs of females. Based on its strong immunogenic properties, a recombinant TcUBQ-2 (rTcUBQ-2)-based ELISA was established and exhibited a sensitivity of 100% and a specificity of 95.8% to detect anti-*T. canis* mouse sera and a sensitivity of 79.2% and a specificity of 83.3% to detect anti-*T. canis* dog sera, respectively. This study presents a comprehensive bioinformatics analysis of the dog roundworm TcUBQ-2, and its strong serodiagnostic performance suggests that rTcUBQ-2 has the potential to be developed into an ELISA-based serological test for detecting toxocariasis in dogs and other accidental hosts, including humans.

## 1 Introduction

With dogs increasingly becoming integral members of the human family, a large number of dog-related pathogens are emerging in the disease spectrum, affecting humans and posing serious threats to public health (1–3). Among these, *Toxocara canis*, the cause of toxocariasis, is emerging as an important yet neglected helminthic zoonosis due to severe or fatal larval migrans in both animals and humans (4–7). Generally, *T. canis* parasitizes dogs and leads to anemia, emaciation, and malnutrition in infected animals (primarily due to intestinal adults). However, humans are accidental hosts and can become infected through the accidental ingestion of infective eggs from the environment or from materials contaminated with the feces of infected animals (4). Following ingestion, the infective third-stage (L3) larvae hatch, penetrate the intestinal wall, and migrate through various organs, triggering severe clinical syndromes in humans, such as ocular larva migrans (OLM), visceral larva migrans (VLM), neurological larva migrans (NLM), and covert toxocariasis (CT) (8, 9). It is estimated that human toxocariasis results in 51751.4 DALYs (disability-adjusted life years) and incurs an economic impact of $2.5 billion annually across 28 countries, including Japan, Denmark, Austria, Sweden, and the US (10). Therefore, toxocariasis has also been classified as one of five neglected parasitic infections by the Centers for Disease Control and Prevention (CDC) and is prioritized for public health initiatives in North America (11).

Current diagnosis and identification of toxocariasis rely on fecal egg examination (mainly in dogs) and histopathological larval confirmation (mainly in humans) (7, 12). However, both methodologies require professional morphological skills and become increasingly challenging when identifying and differentiating eggs or larvae among various potential environmental cross-contaminating eggs from other parasites, including morphologically similar *Toxocara cati* and *Toxocara malaysiensis* (13, 14), as well as potentially contaminating nematode larvae from *Baylisascaris* spp., *Ascaris* spp., *Angiostrongylus* spp., and *Ancylostoma* spp. (15, 16). Therefore, obtaining an efficient and reliable method to identify and differentiate *T. canis* eggs or larvae has become crucial for clinical diagnosis, epidemiological investigation, and laboratory tests, and achieving this goal seems feasible only through the utilization of molecular methodologies. Indeed, several molecular PCR methods that use the ribosomal ITS and mitochondrial cox1 and nad1 as markers to detect *T. canis* have been developed for this purpose (17). These methods, however, cannot diagnose migrating larvae or adults outside the egg-laying period. Hence, an alternative, more efficient molecular method is still needed. Serodiagnosis, particularly ELISA tests (enzyme-linked immunosorbent assays) equipped with target molecules that play excretory/secretory (E/S) roles and functions in the survival, development, and immune evasion of parasites (18), represents an ideal and preferable strategy. Until now, several native *T. canis* ES proteins (ESPs)-based ELISAs have been experimentally proven to possess good diagnostic sensitivity, but their cross-reactivities with antibodies to other intestinal parasites and labor-intensive collections pose significant limitations for widespread clinical practice (12, 19–21).

The ubiquitin-proteasome system (UPS) is a major intracellular and non-lysosomal protein degradation system involved in cell differentiation and proliferation in eukaryotic cells (22, 23). This system has also been identified in nematodes, where its two key components, polyubiquitin (UBQ-1) and ubiquitin plus an L40 ribosomal protein (UBQ-2), have been further confirmed to participate in the meiotic divisions of the free-living nematode *Caenorhabditis elegans* (24, 25). The silencing of Ceubq-2 induces high larval mortality (26). In parasitic nematodes, *Heterodera schachtii* UBQ-like is hypothesized to relate to parasite–host interactions, especially during syncytium formation (27). *Strongyloides venezuelensis* UBQ is proposed to regulate nematode development (28), while *Haemonchus contortus* UBQ has been shown to play an important role in the worm’s survival during desiccation (29). Additionally, its UBQ-2 is considered essential for the development of this parasite (30). Consequently, some researchers suggest that parasite-derived UBQ-2, including that of parasitic nematodes, could be selectively targeted for drug intervention and diagnostic purposes. Given that no information on UBQ-2 of *T. canis* is available thus far and that *T. canis*-specific immunogenic proteins as diagnostic agents are lacking, the aims of this study are to (i) clone a new ubq-2, Tcubq-2, from *T. canis*; (ii) analyze its biological characteristics; and (iii) test the immunogenicity and preliminary ELISA-based diagnostic potential of its recombinant protein TcUBQ-2 (rTcUBQ-2) in mice and dogs using naïve and infected sera. The results of this work will provide the foundation for the development of TcUBQ-2 as a candidate serodiagnostic antigen to detect toxocariasis.

## 2 Materials and methods

### 2.1 Animals and parasites

A total of 58 specific pathogen-free (SPF) BALB/c mice (females, 6 weeks old), 48 healthy Chinese Kunming dogs (body weights: 10–15 kg, 3 months old), and three healthy beagles (body weights: 4.5 ± 0.5 kg, 3–4 months old, born in the same litter) were purchased from Dashuo Experimental Animal Co. Ltd. (Sichuan, China). *T. canis* male adults, female adults, fourth-stage (L4) larvae, and fifth-stage (L5) larvae in the present study were harvested from the intestinal tracts of euthanized, naturally infected dogs in Wenjiang, Sichuan Province, China. Embryonated eggs of *T. canis* were obtained from the uteri of female adults and then incubated in 2.5% formaldehyde at 28°C with 80% relative humidity for 28 days to collect the infective egg-containing third-stage (L3) larvae, which were then stored in a 2.5% formalin solution at 4°C until use (31). Three beagles were orally inoculated with 5,000 infective eggs each, and their intestine-hatched L3 and lung-migrating L3 larvae were collected at different times after infection using the agar-gel method (32, 33). In addition, the phosphate-buffered saline (PBS)-soluble parasite proteins from these developmental stages of *T. canis* were isolated using the ExKine™ Pro Total Protein Extraction Kit (Abbkine, Wuhan, China) and quantified using the micro-bicinchoninic acid (BCA) protein assay kit (Pierce/Thermo Fisher Scientific, Asheville, NC, United States).

### 2.2 RNA isolation and cDNA synthesis

Total RNA was extracted from *T. canis* adults using TRIzol reagent (Invitrogen, Carlsbad, United States) and subsequently reverse-transcribed into first-strand cDNA with the Revert Aid First Strand cDNA Synthesis Kit (Thermo Fisher Scientific, Waltham, MA, United States) in accordance with the manufacturer’s instructions. Specifically, Oligo (dT) 18 Primer, DEPC-treated water, 5 × reaction buffer, RNase Inhibitor, dNTP mix, AMV Reverse Transcriptase, and total RNA were used to obtain first-strand cDNA using the RT-PCR program: denaturation at 42°C for 60 min, followed by 72°C for 5 min, and extension at 8°C for 5 min. The cDNA was stored at −20°C until further use.

### 2.3 Cloning of Tcubq-2 gene

The full-length cDNA sequence of the Tcubq-2 gene was amplified by PCR using primers containing the restriction enzyme sites *Bam*HI and *Xho*I, as shown in Table 1. The PCR reaction system consisted of 25 μL, including 1.5 μL of cDNA (approximately 50 ng/μL), 1.5 μL of each primer (forward and reverse, 10 pmol), 8 μL of ddH_2_O, and 12.5 μL of 2 × *Taq* Plus Master Mix (0.1 U *Taq* Platinum Polymerase/μL, 500 μM dNTP each, 20 mM Tris–HCl, 100 mM KCl, three mM MgCl_2_; TIANGEN, Beijing, China). The PCR amplification procedure was as follows: pre-denaturation at 95°C for 5 min, followed by 35 cycles at 95°C for 30 s, 51°C for 30 s, and 72°C for 30 s, then 72°C for 5 min, and finally storing at 8°C. The PCR products were purified using the TIANgel Midi Purification Kit (TIANGEN), cloned into the pMD19-T vector (TaKaRa, Dalian, China), and then transformed into *Escherichia coli* DH5α cells (TIANGEN). Then, the positive clones were selected and sequenced by Chengdu TSINGKE Biotech Company (Sichuan, China). The correct sequence of the Tcubq-2 gene has been submitted to the GenBank database under the accession no. PQ778081.

**Table 1:** Primer information.

Primer	Primer sequences (5′ to 3′)	Annealing temperature/°C	Product size/bp	Utilization
Tcubq-2	F1: CGCGGATCCATGCAGATTTTTGTGAAGACTC R1: CCGCTCGAGTCACTTCAGCTTCTTTTTAACG	51	387	Cloning/RT-PCR
18S	F2: GGTTATATGCTTATCTCAAAGGCTAA R2: GGAAACCTTGTTACGACTTTTGC	58	1,754	RT-PCR
Tcubq-2	F3: TTTTCGCCGGAAAGCAGTTG R3: ATTCCACCCCTCAATCGCAG	60	100	qPCR
18S	F4: AATTGTTGGTCTTCAACGAGGA R4: AAAGGGCAGGGACGTAGTCAA	60	137	qPCR

### 2.4 Bioinformatics analysis of TcUBQ-2

To explore the biological characteristics of TcUBQ-2, the open reading frame (ORF) and deduced amino acid sequence of this gene were obtained using the ORF Finder and the Lasergene software package for Windows (DNASTAR, Madison, WI, United States) and were then assessed with ExPASy online servers. Among these analyses, the isoelectric point (pI) and molecular weight (Mw) of TcUBQ-2 were calculated using the Compute pI/Mw tool; physicochemical parameters were analyzed using ProtParam; the signal sequence was inferred using SignalP ver. 4.0; the transmembrane motif was predicted using TMHMM ver. 2.0, and post-translational modifications were predicted using FindMod. The secondary (2D) structure was predicted using NetSurfP (34), while the tertiary (3D) structure was predicted using AlphaFold3 with default parameters, followed by stereochemical quality validation using PROCHECK to generate the Ramachandran plot as described elsewhere (35). Based on these biological characteristics, a multiple sequence alignment was conducted using ClustalW2 and the online BLASTp tool, with the conserved sequence logos in the alignment constructed and visualized using WebLogo 3, followed by a neighbor-joining (NJ)-based phylogenetic analysis using MEGA 11.0 (36, 37). A list of all bioinformatics analysis software and its applications included here can be found in Table 2.

**Table 2:** Software and applications for bioinformatics analysis.

Software	Websites	Applications
ORF finder	https://www.ncbi.nlm.nih.gov	Open reading frames prediction
TMHMM Server 2.0	https://www.cbs.dtu.dk/services/TMHMM-2.0/	Transmembrane areas prediction
SignalP Server 6.0	https://www.cbs.dtu.dk/services/SignalP-6.0/	Signal peptide prediction
ExPASy ProtParam Server	https://web.expasy.org/protparam/	Analysis of physical and chemical properties
NetSurfP - 3.0	https://services.healthtech.dtu.dk/services/NetSurfP-3.0/	2D structure prediction
AlphaFold 3.0	https://deepmind.google/technologies/alphafold/alphafold-server/	3D structure prediction
BLASTp	https://blast.ncbi.nlm.nih.gov/Blast.cgi?PROGRAM=blastp&PAGE_TYPE=BlastSearch&LINK_LOC=blasthome	Protein sequence comparisons
WebLogo 3	https://weblogo.threeplusone.com/	Sequence logo analysis

### 2.5 Transcription analysis of TcUBQ-2

In parallel with bioinformatics analyses of TcUBQ-2, we investigated the transcriptional levels of TcUBQ-2 throughout the lifecycle of *T. canis*. Embryonated eggs and various developmental stages of worms (egg-containing L3 larvae, EL3; intestine-hatched L3 larvae, IL3; lung-migrating L3 larvae, LL3; L4–L5 larvae, L4–L5; female adults, FA; male adults, MA) were subjected to RNA isolation, followed by cDNA synthesis as previously described (38). RT-PCR was used to detect the Tcubq-2 gene at these developmental stages, with the ribosomal 18S gene (GenBank accession no. U94382) used as the positive control. Additionally, transcription levels of TcUBQ-2 at these eight stages were quantified using real-time quantitative PCR (qPCR) on a LightCycler 96 real-time fluorescent quantitative PCR system (Bio-Rad, California, United States). The qPCR primers for the Tcubq-2 gene are listed in Table 1. The reaction system consisted of 20 μL, which included 10 μL of 2 × SuperFast Universal SYBR Master Mix (Cwbio, Jiangsu, China), 0.4 μL of each primer, 1 μL of cDNA, and 8.2 μL of ddH_2_O. The reaction conditions were set to 95°C for 30 s, followed by 40 cycles of 95°C for 5 s and 60°C for 30 s, 95°C for 15 s, 95°C for 1 min, and 60°C for 15 s. Each sample was tested in triplicate. The transcriptional levels were normalized to the mRNA level of the internal reference ribosomal 18S gene using the comparative Ct method (2^−∆∆Ct^) (39).

### 2.6 Expression and purification of rTcUBQ-2

The PCR products with the correct sequence of Tcubq-2 were ligated into the pET32a (+) vector (TaKaRa), and the recombinant pET32a (+)-Tcubq-2 was transferred into *E. coli* Rosetta cells (Invitrogen). Positive transformants were grown overnight in Luria-Bertani (LB) broth containing 100 μg/mL ampicillin at 37°C until the optical density (OD) at 600 nm reached approximately 0.4–0.6. The expression of TcUBQ-2 was induced using 1 mM isopropyl β-D-thiogalactopyranoside (IPTG) at 37°C for 6 h. Induced cells were centrifuged at 12,000 rpm for 1 min, and the supernatant was discarded. The cell sediment was then lysed using sonication in an ice-water bath. The supernatants and precipitates were separately analyzed using SDS-PAGE to determine the expression form of the TcUBQ-2 protein. The recombinant TcUBQ-2 (rTcUBQ-2) protein was purified using Ni-NTA resin columns (Bio-Rad), according to the manufacturer’s protocol, and stored at −80°C until further use.

### 2.7 Sera preparations

To produce specific anti-rTcUBQ-2 polyclonal antibodies, 10 BALB/c mice were subcutaneously injected with 50 μg of purified rTcUBQ-2 emulsified with Freund’s complete adjuvant (Sigma, St. Louis, United States). This was followed by two booster immunizations using the same dose but emulsified with Freund’s incomplete adjuvant (Sigma) at fortnightly intervals. Mice were bled 1 week after the last immunization for serum collection, and the pooled anti-rTcUBQ-2 sera were stored at −20°C until use (38). In addition, anti-*T. canis* sera from mice (*n* = 24) and dogs (*n* = 24) were separately collected from artificially infected BALB/c mice and Chinese Kunming dogs, as described elsewhere (20, 40). Dog immune sera against the co-infected endoparasite representatives *Ancylostoma caninum* (*n* = 6) and *Dipylidium caninum* (*n* = 6) were provided by the Department of Parasitology, College of Veterinary Medicine, Sichuan Agricultural University.

### 2.8 Western blotting analysis

Western blotting analysis was conducted as previously described (15). Briefly, the parasite proteins and purified rTcUBQ-2 were lysed in an electrophoresis sample buffer, subjected to 12% SDS-PAGE, transferred onto nitrocellulose membranes, and blocked with 5% non-fat milk in PBS buffer for 2 h. Parasite-derived TcUBQ-2 was detected by incubating the membranes with mouse anti-rTcUBQ-2 serum. To assess the antigenicity of rTcUBQ-2, mouse sera from animals inoculated with *T. canis* infective eggs and naïve mouse sera were used. Additionally, the HRP-conjugated mouse anti-His-Tag monoclonal antibody (mAb) (Abclonal, Wuhan, China) was included as a positive control. After incubating with these primary antibodies overnight at 4°C, each membrane was further incubated with 1:2000 diluted HRP-conjugated goat anti-mouse IgG (Abclonal) at room temperature for 1 h. The membranes were washed four times with Tris-Buffered Saline Tween-20 (TBST) for 5 min between each step. Finally, the protein bands were visualized using 3,3′-diaminobenzidine tetrahydrochloride (DAB) (TIANGEN) or enhanced chemiluminescence (ECL) (Sigma) (39).

### 2.9 Immunofluorescence assay

The immunofluorescence assay (IFA) was performed as previously described (41). Briefly, the adult male and female *T. canis* in the host gut were fixed in 4% paraformaldehyde for 24 h, embedded in paraffin blocks, and sectioned into 5-μm histological slices. Subsequently, the sections were deparaffinized with dimethylbenzene, dehydrated through graded concentrations of ethanol, and heated in 0.01 mol/L sodium citrate buffer (pH 6.0) for 10 min for antigen retrieval. After three washes with PBS, the sections were blocked with 5% bovine serum albumin and sequentially incubated with a 1:100 dilution of anti-rTcUBQ-2 mouse sera or naïve mouse sera, followed by a 1:200 dilution of fluorescein isothiocyanate (FITC)-conjugated goat anti-mouse IgG (Abclonal); sections inoculated solely with the same secondary antibody served as controls. The stained samples were mounted in glycerol/phosphate buffer (v/v, 9:1) and examined under a fluorescent microscope (BX53; Olympus, Tokyo, Japan).

### 2.10 rTcUBQ-2-based ELISA

Prior to the rTcUBQ-2-based ELISA tests, a checkerboard titration test was conducted to identify the optimal dilutions of rTcUBQ-2, tested sera, and secondary antibodies. In brief, polystyrene 96-well microplates (Invitrogen) were coated with 100 μL per well of rTcUBQ-2 diluted in carbonate/bicarbonate buffer (pH 9.6) at a serial two-fold dilution ratio (ranging from 1:40 to 1:1280) and incubated overnight at 4°C. A 5% non-fat milk solution diluted in 1 × PBS was used to block each well at 37°C for 1 h, after which 100 μL of serum samples, diluted as previously mentioned in PBS, were added to the plates. After 1 h of incubation at 37°C, 100 μL of goat anti-mouse/dog IgG-HRP (Abclonal) conjugate diluted at three different ratios (1:5000, 1:10000, and 1:15000) in PBS was added to each well and incubated for an additional hour at 37°C. The plates were washed three times with PBS containing 0.5% Tween-20 (PBST) after each incubation step. Finally, the antibody–antigen reactions were developed with 100 μL of tetramethylbenzidine (TMB) at room temperature in the dark and terminated with 100 μL of stop solution (2 M H_2_SO_4_) after 20 min. The optical density (OD) of each well was measured at a wavelength of 450 nm (OD_450_) using the ELISA spectrophotometer (Thermo Fisher Scientific). The P/N value was calculated as OD_450_ of positive sera divided by OD_450_ of negative sera for subsequent experiments. The OD_450_ values of naïve serum samples were collected under the optimal conditions mentioned above to determine the cut-off value, which was defined as the mean OD_450_ value +3 × standard deviations (42).

The OD_450_ value for 48 infected serum samples (24 from mice and 24 from dogs) and 48 naïve serum samples (24 from mice and 24 from dogs) was measured under optimal conditions and identified as positive or negative by comparison with the cut-off value. Sensitivity and specificity were calculated using the following formulas: sensitivity = (ELISA test positive/true *T. canis* positive) × 100%, and specificity = (ELISA test negative/true *T. canis* negative) × 100% (42), respectively. The results obtained from both mouse and dog serum samples were further subjected to receiver operating characteristic (ROC) analysis using IBM SPSS statistical software to evaluate their accuracy, specifically the area under the ROC curve (AUC). In addition, 12 heterosera derived from dogs infected with *A. caninum* and *D. caninum* were included for cross-activity evaluation. To assess the stability of the ELISA established here, three separate batches, each containing eight negative mouse or dog sera, were tested. Each serum sample was analyzed in triplicate, and the OD_450_ value was measured three times on different ELISA plates. The coefficient of variation (CV) was calculated by determining the standard deviation and average to assess repeatability between plates.

### 2.11 Statistical analysis

ELISA data were expressed as mean ± standard deviation. Statistical differences between the test sera groups were assessed using one-way analysis of variance (ANOVA) with SPSS version 17.0 for Windows (SPSS Inc., Chicago, IL), and *p*-values of <0.05 were considered statistically significant.

## 3 Results

### 3.1 Molecular characterization of TcUBQ-2

#### 3.1.1 Sequence characteristics

**Figure 1:**
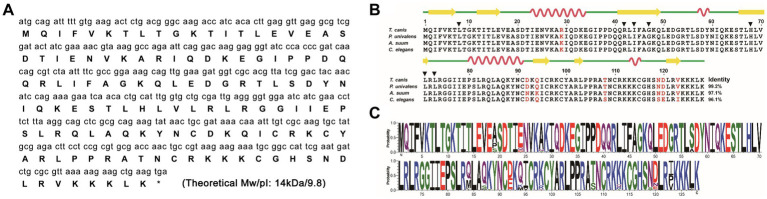
Bioinformatics analysis of TcUBQ-2. **(A)** The ORF and deduced amino acid sequence of TcUBQ-2 are presented. The stop codon is marked with an asterisk. The predicted molecular weight (Mw) and isoelectric point (pI) are indicated at the end of the polypeptide. **(B)** Sequence alignment and secondary structure modeling of TcUBQ-2 with nematode homologs. For the alignment, the following sequences were retrieved from the PDB database (species and accession numbers are indicated in parentheses) and aligned using the ClustalW2 program: TcUBQ-2 (*Toxocara canis*; PQ778081), PuUBQ-2 (*Parascaris univalens*; P46436), AsUBQ-2 (*Ascaris suum*; Q9NAW7), and CeUBQ-2 (*Caenorhabditis elegans*; P46436). Sites of weak similarity are shown in red. Percentages of sequence similarity with respect to TcUBQ-2 are shown at the C-terminus. The inferred seven amino acid positions that comprise the binding interface of TcUBQ-2 are indicated by black triangles. For the secondary structure of TcUBQ-2, the elements, including strands and helices, are shown above the alignment as yellow arrows and red loops, respectively. **(C)** Sequence logo of UBQ-2. The x-axis represents the amino acid residue position from the N to C termini. Letter sizes indicate the residue frequency at a given position among all repeats. The letter colors indicate the side residue charge [black, hydrophobic; red, negative charge; blue, positive charge; green, polar (without amides); purple, polar (with amides)].

The novel ubiquitin 2, Tcubq-2, was initially identified through homologous screening of the *T. canis* transcriptome dataset. The full-length cDNA of Tcubq-2 was 387 bp and encoded a polypeptide of 128 amino acids, with a predicted molecular weight of 14 kDa and a pI of 9.8 (Figure 1A). ProtParam analysis indicated a high proportion (70.8%) of hydrophilic amino acid residues in TcUBQ-2, suggesting its hydrophilicity. SignalP prediction showed a lack of signal peptide at the N-terminal of TcUBQ-2, and TMHMM analysis indicated that TcUBQ-2 lacked transmembrane segments (Supplementary Figure S1). Similarly, no post-translational modifications were found in TcUBQ-2 based on the results from ExPASy-FindMod.

#### 3.1.2 Multi-sequence alignment

**Figure 2:**
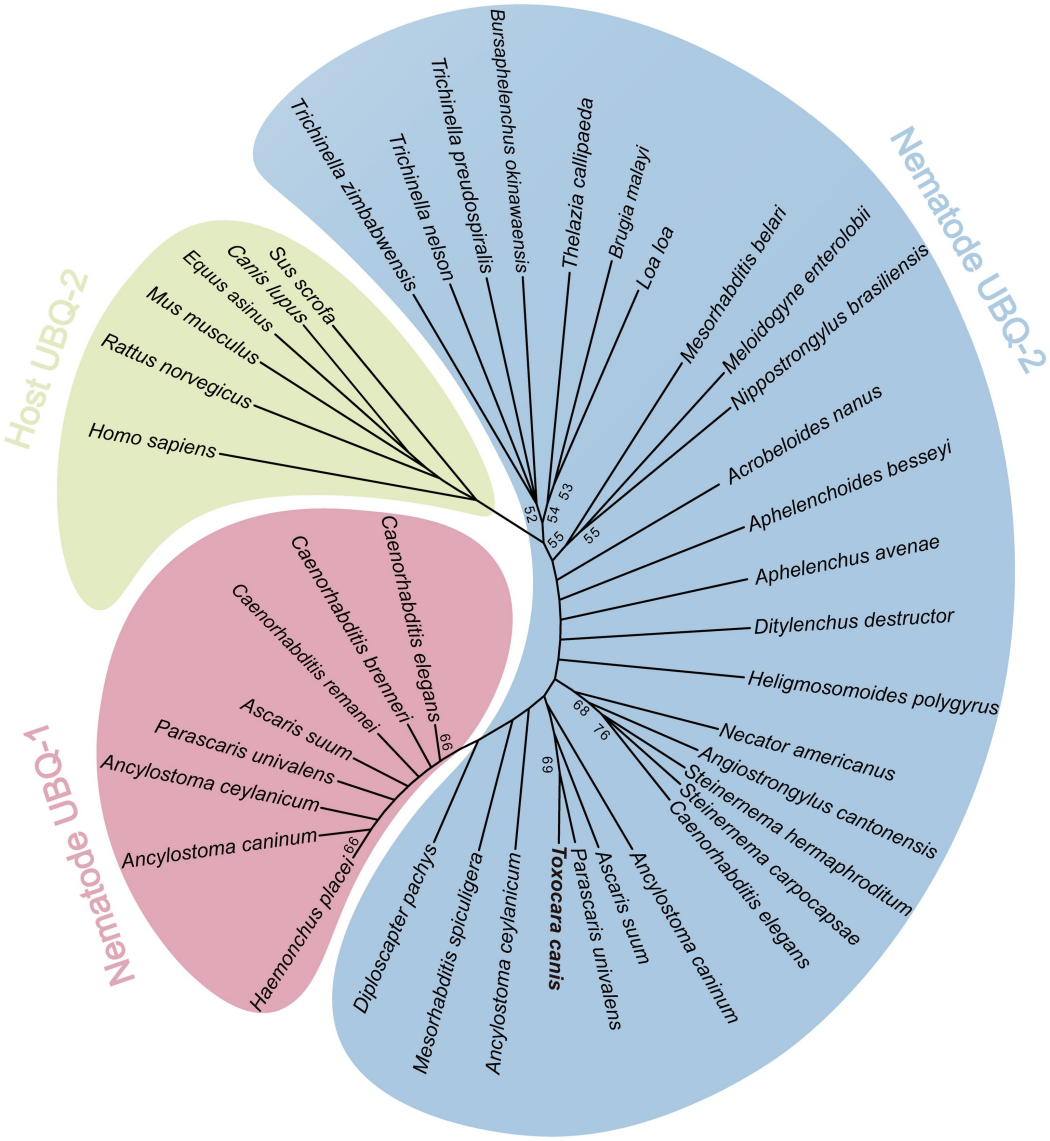
Phylogenetic relationships of TcUBQ-2 and other ubiquitins from nematodes and roundworm hosts. An unrooted phylogenetic tree was inferred by neighbor-joining (NJ) analysis in MEGA11. The tree was constructed from an amino acid sequence alignment using ClustalW2. By means of the GTR + I + G model, 1,000 bootstrap replicates were run to calculate the percentage reliability for each node, and only values ≥50% are shown. The organism species from which the UBQ protein sequences are retrieved from GenBank or UniProt and used in the tree are listed as follows: nematode UBQ-1: *Caenorhabditis elegans* (AAA28154.1), *C. brenneri* (G0MLK4), *C. remanei* (E3LYQ5), *Ascaris suum* (F1L201), *Parascaris univalens* (A0A914ZTG3), *Ancylostoma ceylanicum* (A0A016WN01), *A. caninum* (A0A368GWN1), *Haemonchus placei* (A0A0N4WVU4); nematode UBQ-2: *Trichinella pseudospiralis* (KRY71437.1), *T. zimbabwensis* (KRZ09314.1), *T. nelsoni* (KRX18586.1), *Bursaphelenchus okinawaensis* (A0A811LPJ3), *Thelazia callipaeda* (VDN02338.1), *Brugia malayi* (CDP91858.1), *Loa loa* (XP_020306345.1), *Mesorhabditis belari* (CAJ0935984.1), *Meloidogyne enterolobii* (CAD2150586.1), *Nippostrongylus brasiliensis* (WKY01473.1), *Acrobeloides nanus* (A0A914E8V7), *Aphelenchoides besseyi* (KAI6173945.1), *A. avenae* (KAH7729084.1), *Ditylenchus destructor* (KAI1708272.1), *Heligmosomoides polygyrus* (VDP34586.1), *Necator americanus* (XP_013301638.1), *Angiostrongylus cantonensis* (KAE9415103.1), *Steinernema carpocapsae* (TKR68314.1), *S. hermaphroditum* (KAK0417700.1), *C. elegans* (P49632), *A. caninum* (RCN29197.1), *Diploscapter pachys* (PAV73538.1), *A. suum* (F1LDJ7), *P. univalens* (A0A915B8X3), *A. ceylanicum* (A0A016VZV7), *Mesorhabditis spiculigera* (A0AA36C9C7), *Diploscapter pachys* (PAV73538.1) and *T. canis* (PQ778081); roundworms host UBQ-2: *Homo sapiens* (NP_001307950.1), *Rattus norvegicus* (P62986), *Mus musculus* (P62984), *Canis lupus* (P63050), *Equus asinus* (A0A9L0ID46) and *Sus scrofa* (P63053).

A multiple sequence alignment indicated that TcUBQ-2 shared the highest identity (99.2%) with the horse parasite *Parascaris univalens* (UniProt: A0A915B8X3), followed by 97.1% identity with the pig parasite *Ascaris suum* (UniProt: F1L9Q8) and 96.1% identity with the free-living nematode *C. elegans* (UniProt: P49632) (Figure 1B). Further sequence logos confirmed the evolutionary conservation of *T. canis* TcUBQ-2 among these nematode species (Figure 1C). Meanwhile, based on this sequence alignment, an NJ-based phylogenetic tree supports two ubiquitin subgroups among nematodes, namely UBQ-1 and UBQ-2, with both subgroups being distinct from the host-origin UBQs (Figure 2). Within the UBQ-2 subgroup, it was also clear that there was a closer relationship between *T. canis*, *A. suum,* and *P. univalens* compared to other nematodes, consistent with previously published results (43).

#### 3.1.3 2D/3D structures

**Figure 3:**
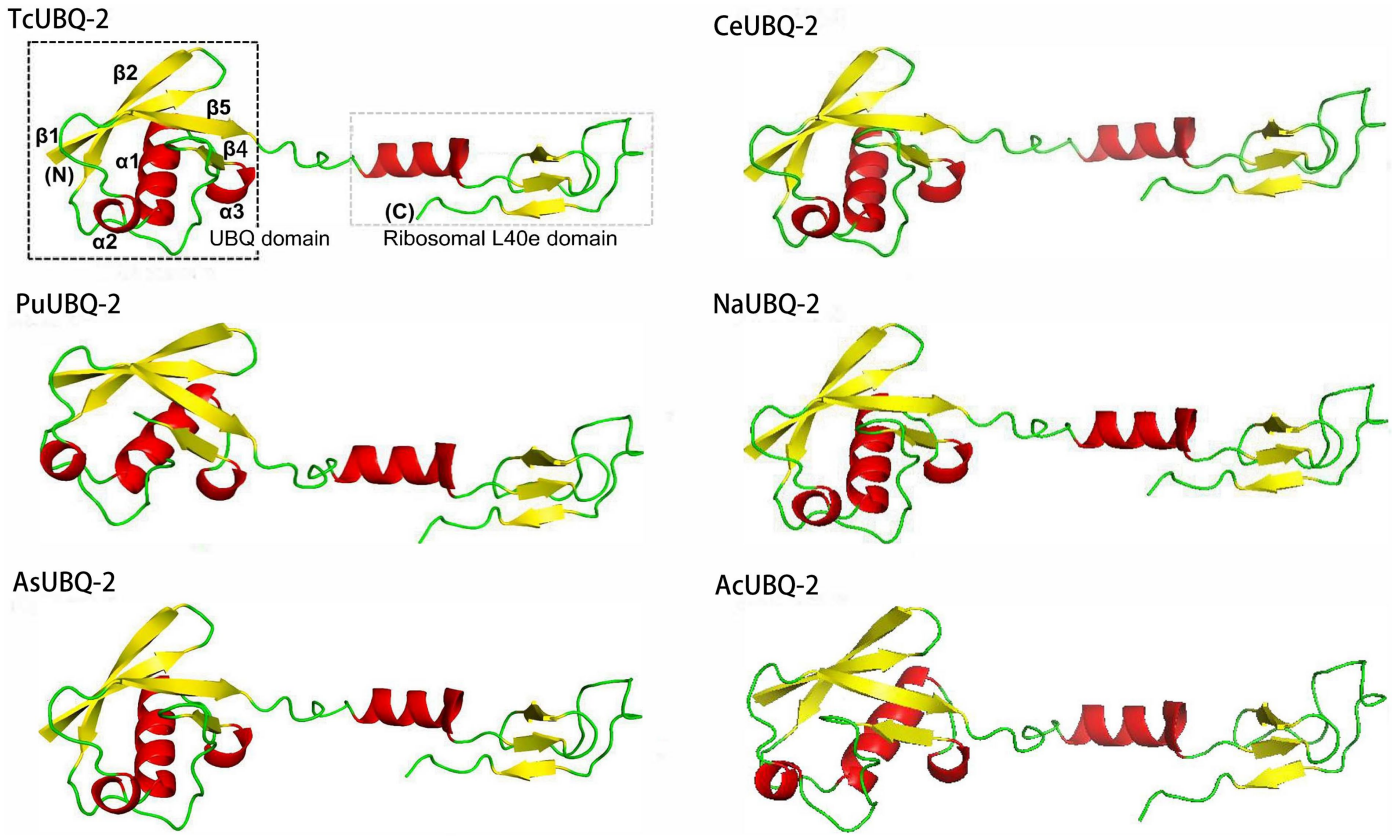
3D structures of TcUBQ-2 and other nematode UBQs. The 3D structures were modeled based on the X-ray structure of *C. elegans* ubiquitin (PDB no.: 1LPL) using AlphaFold (https://alphafold.com/). TcUBQ-2, *T. canis* UBQ-2; PuUBQ-2, *P. univalens* UBQ-2; AsUBQ-2, *A. suum* UBQ-2; CeUBQ-2, *C. elegans* UBQ-2. The N-terminal ribosomal-L40e domain and the C-terminal UBQ domain (including β1, β2, β4, β5, and the α1, α2, and α3 motifs) are shown in TcUBQ-2.

Similar to other nematode homologous ubiquitins, the secondary structure of the TcUBQ-2 protein comprises 27.34% α-helices, 9.38% extended strands, 22.66% β-turns, and 40.62% random coils (Figure 1B). Using this secondary structure alongside the X-ray structure of *C. elegans* ubiquitin (PDB no.: 1LPL), a 3D model of TcUBQ-2 was created and illustrated in Figure 3. Notably, within this typical UBQ domain, there are four β-strands (β1, β2, β4, and β5) arranged against a three-turn α-helix (α1, α2, and α3). It appears that a lack of β3 in TcUBQ-2 is a nematode-specific feature, particularly when compared to other homologs, including those from parasitic hosts.

#### 3.1.4 Transcriptional changes of TcUBQ-2

**Figure 4:**
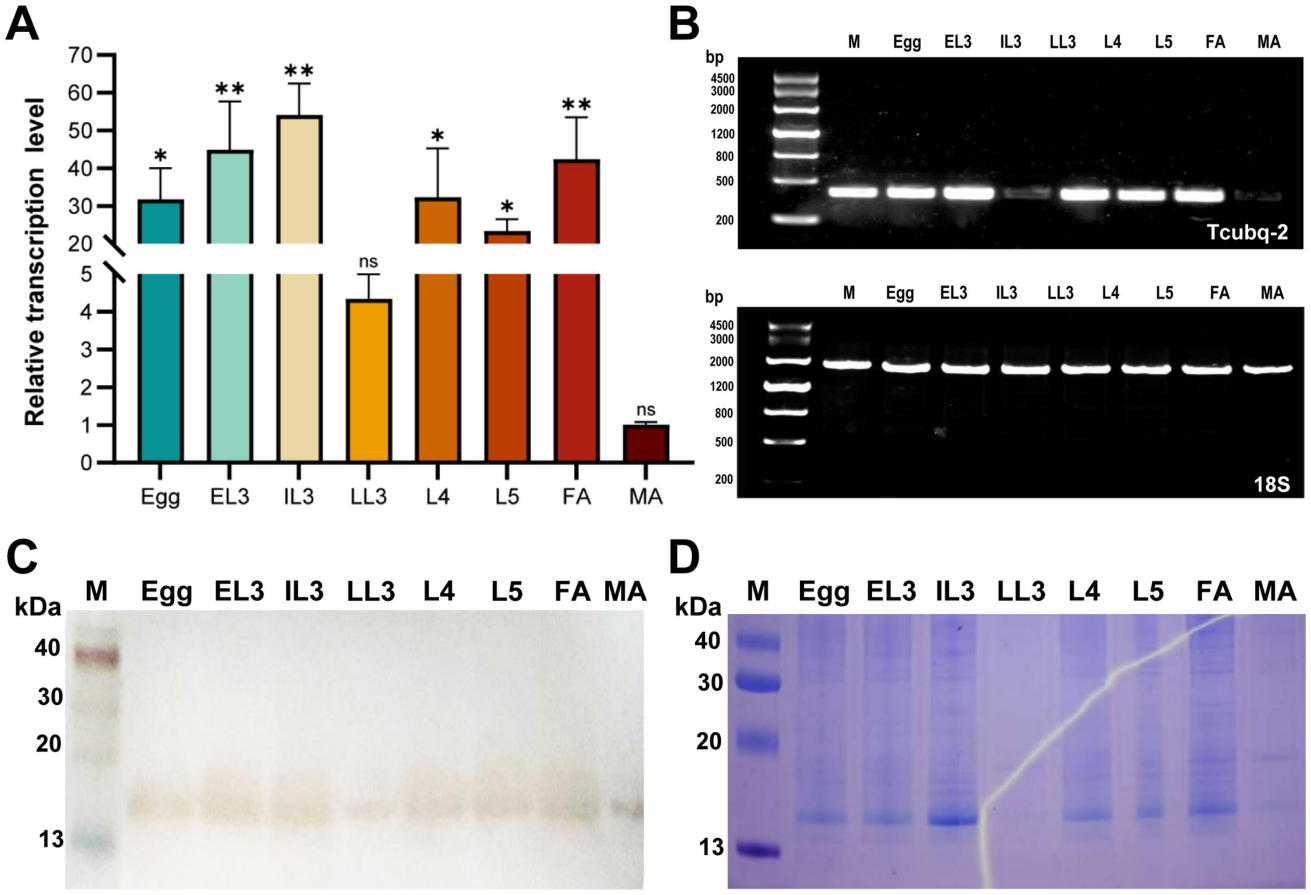
Changes in mRNA and protein expression of TcUBQ-2 throughout the lifecycle of *T. canis*. **(A)** Expression profiles were calculated using the 2^−ΔΔCT^ method and normalized to the levels of 18S ribosomal RNA in *T. canis*. **(B)** RT-PCR analysis of Tcubq-2 transcription, with 18S as the control. **(C)** Western blot analysis of native TcUBQ-2. **(D)** SDS-PAGE analysis of native TcUBQ-2. M, molecular mass marker in kDa; Egg, embryonated eggs; EL3, egg-containing L3 larvae; IL3, intestine-hatched L3 larvae; LL3, lung-migrating L3 larvae; L4-L5, L4-L5 larvae; FA, female adults; MA, male adults. The qPCR data are presented as means and standard deviation (SD) from triplicate experiments. ^*^p < 0.05, ^**^p < 0.01. The “ns” indicates no statistically significant difference.

The mRNA level of the Tcubq-2 gene was analyzed throughout the *T. canis* lifecycle using qPCR. The results showed that Tcubq-2 was transcribed in all eight developmental stages of *T. canis*, with the highest mRNA expression levels observed in the infective IL3 stage (*p* < 0.01), followed by EL3 and FA, and then L4-L5 and embryonated eggs (*p* < 0.05; Figures 4A,B). Conversely, MA exhibited the lowest mRNA level of the Tcubq-2 gene (*p* > 0.05) compared to the internal reference 18S. Similar protein expression patterns of TcUBQ-2 were observed across all eight developmental stages of *T. canis* (Figures 4C,D), suggesting that endogenous TcUBQ-2 is consistently expressed throughout *T. canis* development. However, its higher expression in females compared to males, to some extent, implies a more active role in egg development, in addition to its involvement in meiotic divisions (24, 25).

### 3.2 Expression and purification of rTcUBQ-2

**Figure 5:**
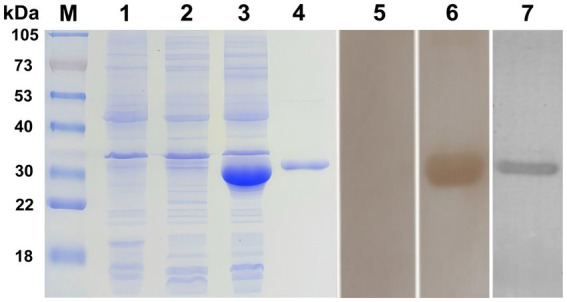
SDS-PAGE and Western blot analysis of purified rTcUBQ-2. M, molecular mass marker in kDa; lane 1, lysate of *E. coli* carrying the pET32a (+) vector; lane 2, lysate of *E. coli* carrying pET32a (+)-Tcubq-2 before IPTG induction; lane 3, lysate of *E. coli* carrying pET32a (+)-Tcubq-2 after IPTG induction; lane 4, purified rTcUBQ-2; lanes 5–7, purified rTcUBQ-2 probed with naïve mouse sera (negative control, lane 5), mouse anti-*T. canis* sera (experimental group, lane 6), and mouse anti-His-Tag mAb (positive control, lane 7), respectively.

The recombinant plasmid pET-32a (+)-Tcubq-2 was successfully expressed in *E. coli* Rosetta as a single His-6-tagged fusion protein, with an expected size of ~34 kDa (lane 3, Figure 5). Due to an additional 20-kDa epitope tag fusion peptide, the molecular mass of rTcUBQ-2 was ~14 kDa, similar to that predicted from its amino acid sequence. Peak expression levels of rTcUBQ-2 were observed at 3 h post-IPTG induction, and expression was detected in both the supernatant and inclusion bodies. To harvest rTcUBQ-2 with a good native structure, the supernatant was purified using Ni–NTA resin columns. After concentration, the purity and yield (~3 mg/L) of rTcUBQ-2 were assessed using SDS-PAGE (lane 4, Figure 5). For western blotting analysis, a positive band at 34 kDa was observed using mouse anti-*T. canis* sera (experimental group) or mouse anti-His-Tagg mAb (positive control), while naïve mouse sera (negative control) showed no reactivity, confirming the good antigenicity of the recombinant protein (lanes 5 vs. 6 and 7, Figure 5).

### 3.3 Immunolocalization of endogenous TcUBQ-2 in adult *Toxocara canis*

**Figure 6:**
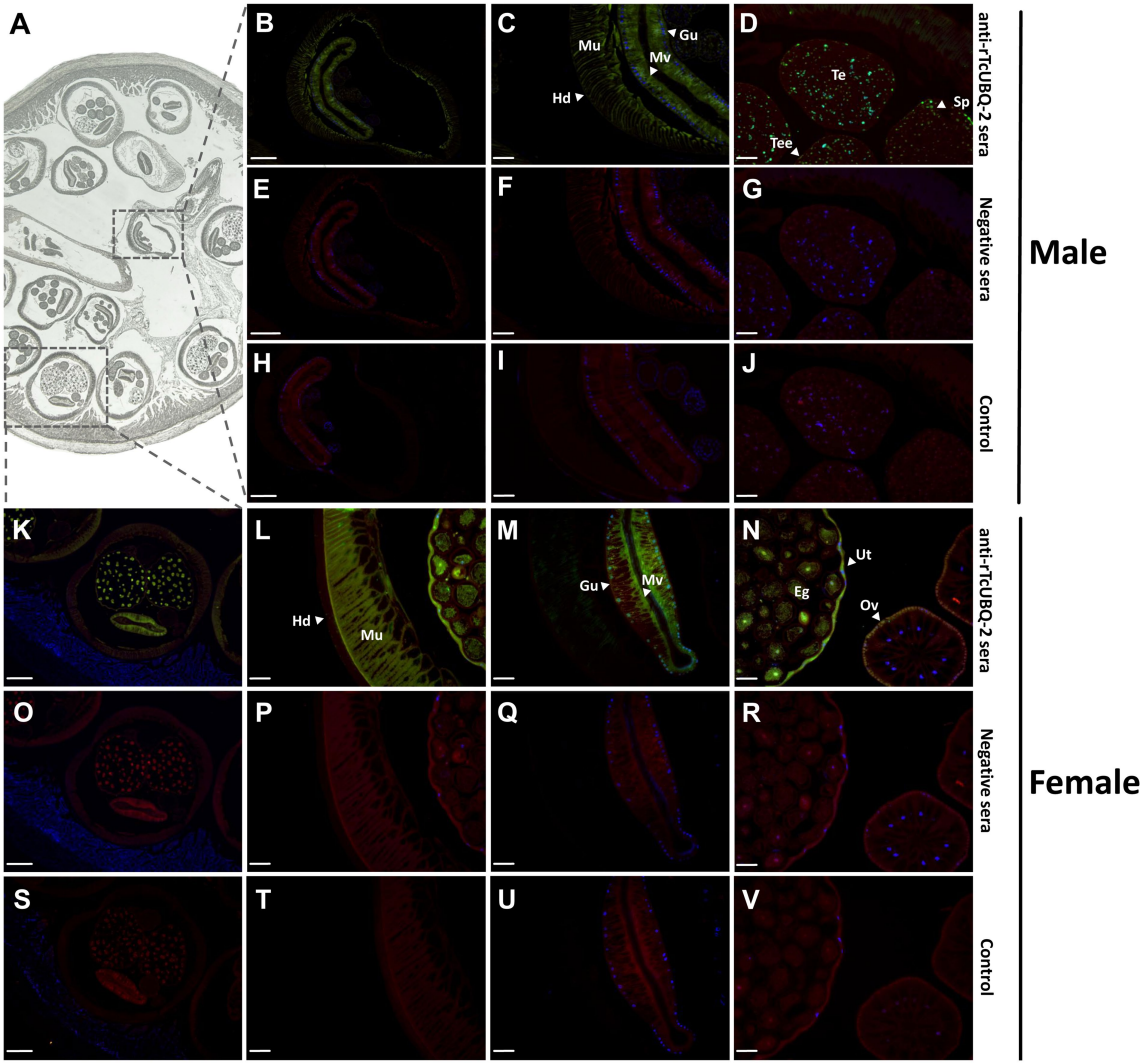
Localization of endogenous TcUBQ-2 in adult *T. canis* using immunofluorescence. **(A)** Sections of *T. canis* within the host dog’s intestine prior to staining. **(B-V)** The sections were incubated with either anti-rTcUBQ-2 mouse serum **(B–D,K–N)**, pre-immune serum **(E–G,O–R)**, or only with the FITC-conjugated secondary antibody **(H–J,S–V)**. White arrows indicate regions labeled by antibodies. Scale bars: **(B,E,H,K,O,S)**; 500 μm; **(C,D,F,G,I,J,L–N, P–R,T–V)**; 50 μm. Hd, hypodermis; Mu, muscle; Gu, gut epithelium; Mv, microvilli; Ov, ovary; Ut, uterus; Eg, egg; Te, testis; Tee, testis epithelium; Sp, sperm.

The tissue localization of endogenous TcUBQ-2 in adult male and female *T. canis* was determined using IFA with anti-rTcUBQ-2 mouse sera and naïve mouse sera (Figure 6A). Specific fluorescence signals were observed in sections probed with anti-TcUBQ-2 sera (Figures 6B–D,K), whereas no fluorescence was detected in sections treated with normal mouse sera (Figures 6E–G,O) or FITC-conjugated secondary antibody only (Figures 6H–J,S). Furthermore, endogenous TcUBQ-2 was mainly located in the hypodermis, muscle tissues, gut epithelium, and microvilli of adult worms (Figures 6B,C,K–M). Notably, the female worm exhibited a stronger fluorescence signal in its reproductive system, including in the uterus, ovary, and non-embryonated eggs, compared to the male reproductive system, such as the testis and sperm cells (Figure 6D vs. Figure 6N). This observation aligns with the mRNA and protein expression analyses of Tcubq-2 in both genders (Figure 4).

### 3.4 rTcUBQ-2-based ELISA

**Figure 7:**
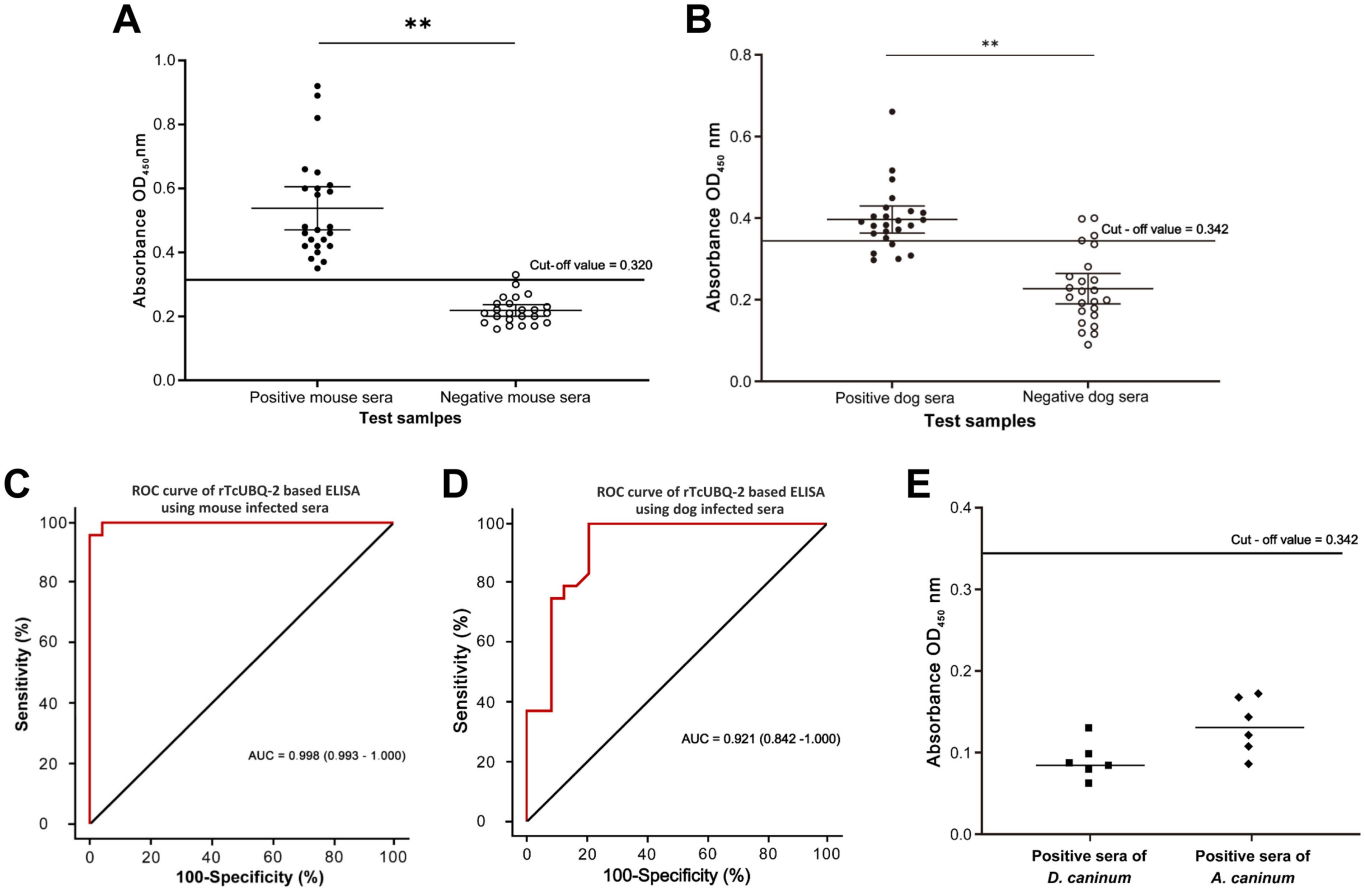
Evaluation of rTcubq-2-based ELISA. **(A)** Sensitivity and specificity of the rTcubq-2-based ELISA in detecting mouse-infected sera. **(B)** Sensitivity and specificity of the rTcUBQ-2-based ELISA in detecting dog-infected sera. The fine horizontal line denotes the critical value for the positive detection cut-off. **(C)** ROC analysis of the rTcUBQ-2-based ELISA using mouse-infected sera. **(D)** ROC analysis of the rTcUBQ-2-based ELISA using dog-infected sera. **(E)** Cross-reactivity of the rTcUBQ-2-based ELISA using heterosera derived from dogs infected with *A. caninum* and *D. caninum*. The fine horizontal line indicates the critical value for the positive detection cut-off (cut-off value = 0.342). ^**^p < 0.01.

Building on the strong antigenicity of rTcUBQ-2, an ELISA-based serodiagnostic approach was developed. After assessing optimal combinations of antigen amounts tested with various dilutions of positive polyclonal sera, a 1:40 dilution (1.7 μg/mL) of rTcUBQ-2 antigen, a 1:80 dilution of sera, and a 1:5000 dilution of the secondary antibody were identified as the best match for the full set of mouse sample tests due to the highest P/N values (Supplementary Table S1). Similarly, a 1:80 dilution (0.85 μg/mL) of rTcUBQ-2 antigen, a 1:40 dilution of sera, and a 1:5000 dilution of the secondary antibody were deemed optimal for the entire set of dog sample tests, owing to its highest P/N values (Supplementary Table S2). Consequently, a cut-off value of 0.320 was finally determined for the TcUBQ-2-based ELISA using mouse sera (Figure 7A), while a cut-off value of 0.342 was established for the TcUBQ-2-based ELISA using dog sera (Figure 7B).

### 3.5 Serodiagnostic potential of rTcUBQ-2-based ELISA

To further assess the serodiagnostic potential of TcUBQ-2-based ELISA, 24 *T. canis*-infected positive mouse sera were detected, with all OD450 values >0.320, corresponding to a sensitivity of 100% (24/24). Similarly, 24 naïve mouse sera were analyzed, and the vast majority (23/24) had OD450 values <0.320, corresponding to a specificity of 95.8%. ROC analysis showed that the AUC for TcUBQ-2-based ELISA was 0.998, indicating excellent discrimination between *T. canis*-positive and negative samples in mice (Figure 7C). Moreover, the intra- and inter-assay CVs for the TcUBQ-2-based ELISA ranged from 2.5 to 14.0% with an average CV of 5.3%, and from 1.6 to 11.3% with an average CV of 4.4%, in mouse samples, respectively (Tables 3, 4), confirming that this test was stable and reproducible.

**Table 3:** The repeatability results of the rTcUBQ-2-based ELISA conducted on a single plate using naïve mouse serum.

Batch	Sample							
	1	2	3	4	5	6	7	8
Batch 1	0.923	0.598	0.597	0.373	0.403	0.424	0.476	0.421
Batch 2	0.816	0.606	0.576	0.348	0.437	0.444	0.472	0.592
Batch 3	0.890	0.661	0.650	0.382	0.445	0.461	0.501	0.484
Mean OD_450_ value	0.876	0.622	0.608	0.368	0.428	0.443	0.483	0.499
Standard deviation	0.045	0.028	0.031	0.014	0.018	0.015	0.012	0.070
Coefficient of variation	5.13%	4.50%	5.10%	3.80%	4.21%	3.39%	2.48%	14.02%

**Table 4:** Repeatability results of rTcUBQ-2-based ELISA using naïve mouse serum across different plates.

Batch	Sample							
	1	2	3	4	5	6	7	8
Batch 1	0.235	0.206	0.243	0.186	0.206	0.213	0.206	0.281
Batch 2	0.227	0.224	0.232	0.173	0.215	0.239	0.221	0.266
Batch 3	0.228	0.224	0.242	0.226	0.214	0.257	0.219	0.258
Mean OD_450_ value	0.230	0.218	0.239	0.195	0.212	0.236	0.215	0.268
Standard deviation	0.004	0.008	0.005	0.022	0.004	0.018	0.007	0.010
Coefficient of variation	1.56%	3.85%	2.09%	11.28%	1.88%	7.62%	3.11%	3.58%

In parallel with the serodiagnostic assessment in mice, 24 *T. canis*-infected dog sera and 24 naïve dog sera were also used to evaluate the TcUBQ-2-based ELISA. Based on the cut-off value of 0.342, 19 infected dog sera were detected as positive, corresponding to a sensitivity of 79.2% (19/24), while 20 samples were detected as negative, yielding a specificity of 83.3% (20/24). Further ROC analysis determined that the AUC for TcUBQ-2-based ELISA was 0.921 (Figure 7D). The intra- and inter-assay average CVs were 3.5 and 6.3%, respectively (Tables 5, 6), confirming that the TcUBQ-2-based ELISA effectively discriminated between *T. canis*-positive and negative serum samples from dogs with good stability and reproducibility. Additionally, both *A. caninum-* and *D. caninumi-*positive dog sera were detected as negative (Figure 7E), ensuring its possible application in detecting toxocariasis in dogs.

**Table 5:** Repeatability results of the rTcUBQ-2-based ELISA using naïve dog sera on a single plate.

Batch	Sample							
	1	2	3	4	5	6	7	8
Batch 1	0.425	0.511	0.382	0.318	0.411	0.393	0.509	0.382
Batch 2	0.409	0.547	0.396	0.327	0.429	0.434	0.513	0.376
Batch 3	0.388	0.501	0.350	0.336	0.448	0.452	0.525	0.396
Mean OD_450_ value	0.407	0.520	0.376	0.327	0.429	0.426	0.516	0.385
Standard deviation	0.015	0.020	0.019	0.007	0.015	0.025	0.007	0.008
Coefficient of variation	3.72%	3.80%	5.12%	2.25%	3.52%	5.79%	1.32%	2.18%

**Table 6:** Repeatability results for rTcUBQ-2-based ELISA using naïve dog sera across different plates.

Batch	Sample							
	1	2	3	4	5	6	7	8
Batch 1	0.271	0.304	0.245	0.274	0.195	0.297	0.223	0.174
Batch 2	0.283	0.284	0.273	0.248	0.241	0.264	0.182	0.209
Batch 3	0.251	0.278	0.244	0.270	0.206	0.282	0.201	0.168
Mean OD_450_ value	0.268	0.289	0.254	0.264	0.214	0.281	0.202	0.184
Standard deviation	0.013	0.011	0.013	0.011	0.020	0.013	0.017	0.018
Coefficient of variation	4.92%	3.85%	5.29%	4.33%	9.16%	4.80%	8.29%	9.84%

## 4 Discussion

*T. canis*-induced toxocariasis is a neglected helminthic zoonosis of global public health significance; however, finding an effective diagnostic tool for detecting *T. canis* infections is still ongoing. In addition to ES antigens, some functional molecules (e.g., HB and RAG1) that contribute to the development and invasion of roundworm parasites are also gaining attention and are considered alternative antigen candidates for developing diagnostic tools (44–46). Similarly, several studies have highlighted the importance of UBQ-2 in the development and immune regulation of parasitic nematodes (26–28, 30). With this in mind, a novel UBQ-2 homologous protein from *T. canis*, referred to as TcUBQ-2, was identified and characterized in this study, where its serodiagnostic potential was validated using indirect ELISA with sera from infected mice and dogs.

After sequencing and conducting a bioinformatics analysis, the ORF of the gene encoding TcUBQ-2 was found to be 387 bp long, which aligns well with the nucleotide range observed in other reported nematodes (24, 29, 47). Peptide structural analyses indicated that TcUBQ-2 exhibited the typical structural features of ubiquitin (UBQ) representatives: four β-strands and a three-turn α-helix, with seven amino acid positions (Leu8, Arg42, Ile44, Gly47, His68, Leu70, and Leu73; Figure 1B) forming the binding interface of TcUBQ-2 (48). Structural biology studies showed that this canonical binding surface of UBQ preferentially interacts with α-helical motifs; however, Gly47 and Leu73 exhibited a stronger preference for interacting with β-strands (48).

Sequence alignment and phylogenetic analysis identified TcUBQ-2 as distinct from host homologs, suggesting its nematode-specific nature (30). Notably, this amino acid diversity indicates its potential as a nematode antigen for diagnostics or therapeutics, given the low likelihood of cross-reactivity with host autoimmune responses. Furthermore, the Tcubq-2 gene was significantly upregulated throughout the lifecycle stages of *T. canis*, especially in the intestine-hatched larvae and adults. Similar expression trends were also observed in our protein analysis (Figures 4C,D), which together not only imply its crucial role in the host invasion and parasitism of *T. canis* but also support our proposal that TcUBQ-2 could be targeted as a suitable diagnostic antigen. Encouragingly, this conclusion was further validated by the immunoblotting assay using its recombinant form, in which rTcUBQ-2 was strongly recognized by the mouse anti-*T. canis* serum and became visible at 34 kDa (Figure 5).

Tissue localization analysis showed that endogenous TcUBQ-2 was present in the hypodermis, muscle, and gut of adult *T. canis*. Within the gut, a strong fluorescent signal for TcUBQ-2 was observed in the brush border of the microvilli, as reported for its homolog in *C. elegans* (49). This finding suggests that UBQ-2 may be involved in metabolism, detoxification, and nutrition, supporting its irreplaceable roles in the development of nematodes (28, 30). In addition, we observed a stronger fluorescent signal of TcUBQ-2 in the ovaries, uterus, and eggs of female *T. canis* compared to that in the male testis and sperm. Whether the expression difference of this gene between genders relates to female-specific reproductive functions remains unknown, but the high expression of TcUBQ-2 in germ cells, including sperm and eggs, implies that TcUBQ-2 might participate in worm meiosis and function in female egg development.

Traditionally, the diagnosis of *T. canis* infection is based on the identification of fecal eggs or worms in dogs, and the infection can only be confirmed after worms are expelled or eggs are shed in the feces (4). Therefore, developing a serodiagnostic tool would strengthen existing diagnostics and improve treatment options, especially during the larval migrating stages. After condition optimizations, a rTcUBQ-2-based ELISA was established here, and our results indicated that this ELISA can accurately detect *T. canis*-specific IgG in experimental mouse sera with a sensitivity of 100% and a specificity of 95.8% (AUC = 0.998 and CV = 5.3 and 4.4%). It also detected *T. canis*-positive dog sera with a sensitivity of 79.2% and a specificity of 83.3% (AUC = 0.921 and CV = 3.5 and 6.3%). Notably, no cross-reactions were observed in *A. caninum-* and *D. caninumi-*positive sera. Such excellent diagnostic performance surpasses that of the previously described two recombinant ES antigens, rTES30 and rTES120 (50), supporting the idea that certain functional molecules from parasites are worthy of consideration as antigen candidates for diagnostic tools.

We did not test for cross-reactions with dog sera from co-infected roundworms, including *Toxocara cati* and *Toxascaris leonina,* because, on the one hand, there has been no information available on the UBQs of these two species to date. On the other hand, it is possible that the rTcUBQ-2-based ELISA cross-reacts with sera from *T. cati* and/or *T. leonina* due to the high evolutionary conservation of UBQ in roundworms (sequence identity >97%; Figures 1B,C). Nevertheless, it is important to note that these parasitic diseases caused by species of *Toxocara* and *Toxascaris* can be effectively treated with the same chemical drugs, such as ivermectin, albendazole, and oxfendazole (16, 51, 52). To sum up, the rTcUBQ-2-based ELISA remains an effective serodiagnostic test for detecting infections with *T. canis* and other related species in dogs.

Finally, given that toxocariasis is a neglected zoonotic disease and its diagnosis often requires a combination of laboratory multi-serological tests (53, 54), it remains prudent to determine when the rTcUBQ-2-based ELISA would be used in clinical practice. Previous studies have shown that the combined use of multiple recombinant antigens increased diagnostic sensitivity and specificity compared to the use of a single antigen (12). For example, three recombinant antigens—rTES-26, rTES-30, and rTES-120—were combined to diagnose human toxocariasis, yielding 100% sensitivity (55). Similarly, two recombinant antigens, Tc-CTL-1 and Tc-TES-26, together achieved 99% sensitivity and 94% specificity in detecting VLM (56). A promising approach to enhance the diagnostic potential of the rTcUBQ-2-based ELISA may involve combining it with other recombinant antigens; however, additional validation studies and the development of a test incorporating rTcUBQ-2 with additional antigens need to be conducted.

## 5 Conclusion

This study characterized a novel *T. canis* ubiquitin, TcUBQ-2, and evaluated its diagnostic potential using its recombinant protein form. The rTcUBQ-2-based ELISA demonstrated strong serodiagnostic potential for detecting *T. canis* infection, regardless of whether mouse or dog-infected sera were used. These findings support its further development as a laboratory test for the diagnosis, epidemiology, and surveillance of toxocariasis in dogs and other accidental hosts, including humans.

## Data Availability

The datasets presented in this study can be found in online repositories. The names of the repository/repositories and accession number(s) can be found in the article/Supplementary material.

## Supplementary material

SUPPLEMENTARY FIGURE S1 Physicochemical properties of TcUBQ-2 and other nematode ubiquitins. Hydrophobicity, signal peptide and transmembrane structure analyses of TcUBQ-2 (*T. canis*, PQ778081) and other nematode ubiquitins, including PuUBQ-2 (*P. univalens*, A0A915B8X3), AsUBQ-2 (*A. suum*, F1LDJ7), AcUBQ-2 (*A. ceylanicum*, A0A016VZV7), NaUBQ-2 (*N. americanus*, XP_013301638.1), CeUBQ-2 (*C. elegans*, P49632).
