# Supplementary figures and images for: Cloning, bioinformatics analysis, and expression of the ubiquitin 2 (ubq-2) gene from the dog roundworm Toxocara canis
Source: Front Vet Sci. 2025 Mar 31;12:1550489. doi: 10.3389/fvets.2025.1550489 (PMC12010969; doi:10.3389/fvets.2025.1550489)

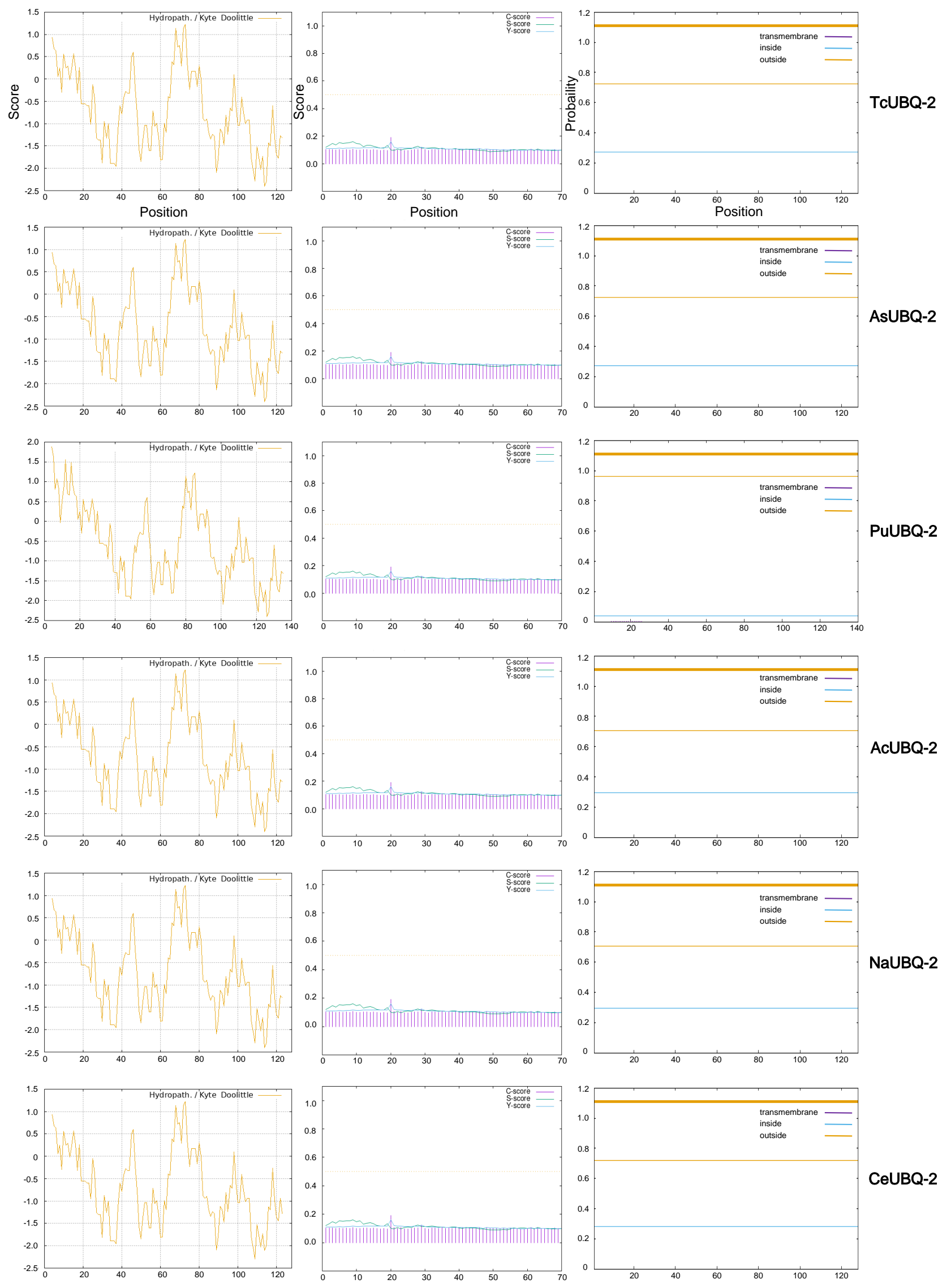

Supplement: SUPPLEMENTARY FIGURE S1 — Physicochemical properties of TcUBQ-2 and other nematode ubiquitins. Hydrophobicity, signal peptide and transmembrane structure analyses of TcUBQ-2 (T. canis, PQ778081) and other nematode ubiquitins, including PuUBQ-2 (P. univalens, A0A915B8X3), AsUBQ-2 (A. suum, F1LDJ7), AcUBQ-2 (A. ceylanicum, A0A016VZV7), NaUBQ-2 (N. americanus, XP_013301638.1), CeUBQ-2 (C. elegans, P49632). [file Supplementary_Image_1.PDF]
