# Supplementary material for: Cloning, bioinformatics analysis, and expression of the ubiquitin 2 (ubq-2) gene from the dog roundworm Toxocara canis
Source: Front Vet Sci. 2025 Mar 31;12:1550489. doi: 10.3389/fvets.2025.1550489 (PMC12010969; doi:10.3389/fvets.2025.1550489)
Supplement: Supplementary file 2 [file Table_1.DOCX]

**Supplementary Table 1. Determinations of the best dilutions of the mouse serum, antigen and secondary antibody in the rTcUBQ-2-based ELISA.**

| **Dilution of sera** | **OD_450_** | | **Dilution of rTcUBQ2** | | | | | | | | |  |
| --- | --- | --- | --- | --- | --- | --- | --- | --- | --- | --- | --- | --- |
|  |  |  | 1:40 | 1:80 | | 1:160 | | 1:320 | 1:640 | | 1:1280 |  |
| **1:40** | P | | 1.117 | 0.961 | | 0.546 | | 0.313 | 0.249 | | 0.251 |  |
|  | N | | 0.468 | 0.409 | | 0.228 | | 0.220 | 0.187 | | 0.171 |  |
|  | P/N | | 2.387 | 2.350 | | 2.395 | | 1.423 | 1.332 | | 1.468 |  |
| **1:80** | P | | 0.963 | 0.843 | | 0.407 | | 0.268 | 0.217 | | 0.211 |  |
|  | N | | 0.347 | 0.342 | | 0.210 | | 0.258 | 0.222 | | 0.219 |  |
|  | P/N | | **2.775** | 2.465 | | 1.938 | | 1.039 | 0.977 | | 0.963 |  |
| **1:160** | P | | 0.753 | 0.673 | | 0.530 | | 0.381 | 0.323 | | 0.317 |  |
|  | N | | 0.387 | 0.309 | | 0.219 | | 0.182 | 0.167 | | 0.172 |  |
|  | P/N | | 1.992 | 2.180 | | 2.420 | | 2.093 | 1.934 | | 1.843 |  |
| **1:320** | P | | 0.616 | 0.642 | | 0.379 | | 0.389 | 0.352 | | 0.330 |  |
|  | N | | 0.306 | 0.296 | | 0.242 | | 0.261 | 0.230 | | 0.233 |  |
|  | P/N | | 2.013 | 2.169 | | 1.566 | | 1.490 | 1.530 | | 1.416 |  |
| **1:640** | P | | 0.447 | 0.407 | | 0.287 | | 0.245 | 0.208 | | 0.257 |  |
|  | N | | 0.262 | 0.243 | | 0.225 | | 0.169 | 0.196 | | 0.143 |  |
|  | P/N | | 1.706 | 1.675 | | 1.276 | | 1.250 | 1.061 | | 1.797 |  |
| **1:1280** | P | | 0.313 | 0.245 | | 0.224 | | 0.210 | 0.188 | | 0.309 |  |
|  | N | | 0.181 | 0.194 | | 0.170 | | 0.160 | 0.174 | | 0.196 |  |
|  | P/N | | 1.729 | 1.263 | | 1.318 | | 1.312 | 1.080 | | 1.577 |  |
| **Dilution of the secondary antibody** | | **P** | | | **N** | | **P/N** | | | **Mean P/N** | | |
| **1:5000** | | 0.669 | | | 0.237 | | 2.823 | | | **2.788** | | |
|  |  | 0.660 | | | 0.227 | | 2.907 | | |  |  |  |
|  |  | 0.645 | | | 0.245 | | 2.633 | | |  |  |  |
| **1:10000** | | 0.408 | | | 0.178 | | 2.292 | | | 2.175 | | |
|  |  | 0.395 | | | 0.203 | | 1.946 | | |  |  |  |
|  |  | 0.462 | | | 0.202 | | 2.287 | | |  |  |  |
| **1:15000** | | 0.188 | | | 0.116 | | 1.621 | | | 1.663 | | |
|  |  | 0.202 | | | 0.114 | | 1.772 | | |  |  |  |
|  |  | 0.201 | | | 0.126 | | 1.595 | | |  |  |  |

Note: P represents positive sera and N represents negative sera.
