# Supplementary material for: Cloning, bioinformatics analysis, and expression of the ubiquitin 2 (ubq-2) gene from the dog roundworm Toxocara canis
Source: Front Vet Sci. 2025 Mar 31;12:1550489. doi: 10.3389/fvets.2025.1550489 (PMC12010969; doi:10.3389/fvets.2025.1550489)
Supplement: Supplementary file 3 [file Table_2.DOCX]

**Supplementary Table 2. Determinations of the best dilutions of the dog serum, antigen and secondary antibody in the rTcUBQ-2-based ELISA.**

| **Dilution of sera** | **OD_450_** | **Dilution of rTcubq2** | | | | | |
| --- | --- | --- | --- | --- | --- | --- | --- |
|  |  | 1:40 | 1:80 | 1:160 | 1:320 | 1:640 | 1:1280 |
| **1:40** | P | 0.807 | 0.734 | 0.555 | 0.472 | 0.383 | 0.297 |
|  | N | 0.337 | 0.281 | 0.229 | 0.204 | 0.179 | 0.165 |
|  | P/N | 2.395 | **2.612** | 2.424 | 2.314 | 2.140 | 1.800 |
| **1:80** | P | 0.671 | 0.495 | 0.470 | 0.412 | 0.356 | 0.286 |
|  | N | 0.297 | 0.264 | 0.247 | 0.197 | 0.167 | 0.138 |
|  | P/N | 2.259 | 1.875 | 1.903 | 2.091 | 2.132 | 2.072 |
| **1:160** | P | 0.458 | 0.374 | 0.361 | 0.299 | 0.240 | 0.197 |
|  | N | 0.192 | 0.189 | 0.171 | 0.178 | 0.119 | 0.106 |
|  | P/N | 2.385 | 1.979 | 2.111 | 1.680 | 2.017 | 1.858 |
| **1:320** | P | 0.402 | 0.266 | 0.243 | 0.217 | 0.198 | 0.197 |
|  | N | 0.161 | 0.127 | 0.101 | 0.102 | 0.095 | 0.098 |
|  | P/N | 2.497 | 2.094 | 2.406 | 2.127 | 2.084 | 2.010 |
| **1:640** | P | 0.305 | 0.267 | 0.249 | 0.224 | 0.226 | 0.197 |
|  | N | 0.143 | 0.138 | 0.139 | 0.110 | 0.090 | 0.102 |
|  | P/N | 2.133 | 1.935 | 1.791 | 2.036 | 2.511 | 1.931 |
| **1:1280** | P | 0.225 | 0.194 | 0.183 | 0.185 | 0.126 | 0.113 |
|  | N | 0.135 | 0.127 | 0.104 | 0.098 | 0.086 | 0.080 |
|  | P/N | 1.667 | 1.528 | 1.760 | 1.888 | 1.465 | 1.413 |

| **Dilution of the secondary antibody** | **P** | **N** | **P/N** | **Mean P/Nf** |
| --- | --- | --- | --- | --- |
| **1:5000** | 0.571 | 0.219 | 2.607 | **2.456** |
|  | 0.591 | 0.240 | 2.463 |  |
|  | 0.563 | 0.245 | 2.298 |  |
| **1:10000** | 0.388 | 0.185 | 2.097 | 2.035 |
|  | 0.361 | 0.168 | 2.149 |  |
|  | 0.372 | 0.200 | 1.860 |  |
| **1:15000** | 0.210 | 0.126 | 1.667 | 1.757 |
|  | 0.204 | 0.113 | 1.805 |  |
|  | 0.196 | 0.109 | 1.798 |  |

Note: P represents positive sera and N represents negative sera.
